# The Siberian centipede species *Lithobiusproximus* Sseliwanoff, 1878 (Chilopoda, Lithobiomorpha): a new member of the Polish fauna

**DOI:** 10.3897/zookeys.821.32250

**Published:** 2019-01-31

**Authors:** Jolanta Wytwer, Karel Tajovský

**Affiliations:** 1 Museum and Institute of Zoology, Polish Academy of Sciences, Wilcza 64, 00-679 Warszawa, Poland Museum and Institute of Zoology, Polish Academy of Sciences Warsaw Poland; 2 Institute of Soil Biology, Biology Centre, Czech Academy of Sciences, České Budějovice, Czech Republic Biology Centre, Czech Academy of Sciences Ceske Budejovice Czech Republic

**Keywords:** Lithobiomorph centipede, new records, western limit of the range, East European Plain

## Abstract

The centipede *Lithobiusproximus* Sseliwanoff, 1878 is presented for the first time as a new member of the Polish fauna. This species, originally characterized as a widespread Siberian boreal species, seems to possess high plasticity with regards to environmental requirements. Its actual distribution range covers several geographical zones where local conditions have allowed it to survive. The present research in the Wigry National Park, northeast Poland, shows that its distribution extends to the ends of the East European Plain embracing the East Suwałki Lake District, where it occurs almost exclusively in the oak-hornbeam forests: in summer it is one of the three dominant lithobiomorph centipedes inhabiting litter layers.

## Introduction

According to data from the catalogue of the Polish centipede fauna (Kaczmarek 1980), Poland has been unevenly explored. Although almost 40 years have passed, there are still areas where the centipede fauna is unknown. The Suwałki region in northeast Poland represents such an area. The present research in the Wigry National Park focuses on natural habitats and evaluation of soil fauna communities and has provided us with the first centipede material related to this area. Included in the material collected, *Lithobiusproximus* Sseliwanoff, 1878 was recorded as a new centipede species for the Polish fauna.

Previously, the chilopod fauna of Poland contained 56 species (Wytwer 2008, Leśniewska et al. 2015); however, a few species found in Poland are widespread and occur also in the east and north of the Palaearctic region, i.e. *Lithobiuscurtipes* C. L. Koch, 1847, *Lithobiuslucifugus* L. Koch, 1862, *Lithobiusforficatus* Linnaeus, 1758, *Geophilusproximus* C. L. Koch, 1847 and *Schendylanemorensis* (C. L. Koch, 1837), but none of them reaches the western limit of its range in Poland.

## Study area, methods, material

The centipede material was collected in forest habitats near the Sobolewo and Krzywe villages in the Wigry National Park, Poland. According to the physicogeographical regionalisation of Kondracki (2002), this area lies within the East Suwałki Lake District mesoregion (Pojezierze Wschodniosuwalskie), the Lituanian Lake District macroregion (Pojezierze Litewskie), the East Baltic Lakeland subprovince (Pojezierze Wschodniobałtyckie), the Eastern Baltic-Belarusian Plain province (Niż Wschodniobałtycko-Białoruski), and the East European Plain megaregion (Niż Wschodnioeuropejski). The deep crystalline basement of the area around the Wigry Lake is created by Precambrian rocks, mainly granites, gneisses, mingmatites, diorites and lamprophyres. The sedimentary cover is represented by sequences of marine or shallow marine Mesozoic and Cenozoic deposits. Sandy soils predominate in most of the area. The Wigry Lake neighborhood is located in the coldest region of Poland (outside the mountains), with a mean annual temperature 6.4 °C and annual precipitation that changes year to year from 330 mm to 830 mm, with a maximum mean in June (87 mm) and minimum in February (25 mm). Snow cover in the last few years has decreased. Winter air circulation is predominated by the western, polar air mass (Rutkowski and Krzysztofiak 2009).

During 2015–2016, surveys were focused on bog-pine forest (*Vacciniouliginosi*-*Pinetum*), bog-birch forest (*Thelypteridi*-*Betuletumpubescentis*), bog-spruce forest (*Sphagnogirgensohni*-*Piceetum*) and alder stands (*Ribesonigri*-*Alnetum*). Since June 2016, it continued in oak-hornbeam forest stands with the *Tilio*-*Carpinetumtypicum* and *Tilio*-*Carpinetumcalamagrostietosumtypicum* plant associations within a running project aimed at assessing the impact of the invasive small balsam *Impatiensparviflora* on forest communities. All stands were located in the Czarna Hańcza River basin.

Centipedes were collected by soil sampling and pitfall trapping. Soil samples (5 samples per plot, sampling area of each 625 cm^2^ and depth 10 cm) were taken twice a year, in bog pine, bog-birch, bog-spruce and alder forests in October 2015 and June 2016, and subsequently in oak-hornbeam forests stands in October 2016 and May and September 2017. Soil samples were transported to the laboratory and invertebrates were subsequently heat extracted using the modified Kempson extraction apparatus (Kempson et al. 1963). Five pitfall traps per plot (cylindrical plastic containers with a volume of 1 L, diameter 10 cm, filled with a killing-preservative water solution of formaldehyde, with detergent and protected with roofs against small vertebrates and rainwater overflow) were exposed continuously during October 2015, June 2016 and October 2016 in bog-pine, bog-birch, bog-spruce and alder forest stands. More extensive sampling (including during October 2016, May 2017 and September 2017) was performed within oak-hornbeam forest stands.

Within the entire centipede material, *Lithobiusproximus* was recorded only in oak-hornbeam forest stands by pitfall trapping. Therefore, the additional data for the following stands relates only to positive records as follows:

Stand 1 *Tilio*-*Carpinetumcalamagrostietosumtypicum*, 54°01'57"N, 22°59'34"E, 167 m a.s.l, forest district 92d, trapping period Oct 2015 – Sept 2017;

Stand 2 *Tilio*-*Carpinetumcalamagrostietosumtypicum*, 54°01'46"N, 23°00'20"E, 158 m a.s.l. forest district 106h, trapping period Oct 2015 – Oct 2016;

Stand 3 *Tilio*-*Carpinetumcalamagrostietosumtypicum*, 54°02'14"N, 23°00'33"E, 169 m a.s.l. forest district 104b, trapping period Oct 2015 – Oct 2016;

Stand 4a *Tilio*-*Carpinetumcalamagrostietosumtypicum*, 54°01'51"N, 23°01'27"E, 154 m a.s.l., forest district 127c, trapping period Oct 2015 – Sept 2017;

Stand 4b *Tilio*-*Carpinetumtypicum*, 54°01'56"N, 23°00'55"E, 154 m a.s.l. forest district 116f, trapping period Oct 2016 – Oct 2017;

Stand 4c *Tilio*-*Carpinetumcalamagrostietosumtypicum*, 54°01'53"N, 23°01'06"E, 153 m a.s.l., forest district 116g, trapping period Oct 2016 – Oct 2017;

Stand 5 *Tilio*-*Carpinetumtypicum*, 54°04'23"N, 23°00'55"E, 149 m a.s.l., forest district 52c, trapping period Oct 2016 – Oct 2017;

Stand 6 *Tilio*-*Carpinetumtypicum*, 54°04'30"N, 23°00'59"E, 161 m a.s.l., forest district 51c, trapping period Oct 2016 – Oct 2017.

The studied material was obtained from the following trapping periods and stands:

15 Jun–26 Sep 2016 – 11 ♀♀, 1 ♂, stand 1; 8 ♀♀, 2 ♂♂, stand 2; 16 ♀♀, 3 ♂♂, stand 3; 3 ♀♀, 3 ♂♂, stand 4a;

26 Sep–19 Oct 2016 – 1 ♀, 1 ♂, stand 2; 1♀, stand 3;

19 Oct 2016–23 May 2017 – 1 ♂, stand 3;

23 May–12 Sep 2017 – 4 ♂♂, stand 4a; 4 ♀♀, 2 ♂♂, stand 4b; 1 ♀, 2 ♂♂, stand 4c; 6 ♀♀, 6 ♂♂, stand 1; 20 ♀♀, 8 ♂♂, stand 5; 8 ♀♀, 8 ♂♂, stand 6.

## Results

### Lithobius (Ezembius) proximus

Taxon classificationAnimaliaLithobiomorphaLithobiidae

Sseliwanoff, 1878

#### Taxonomical remarks.

**Figures 1–5.**
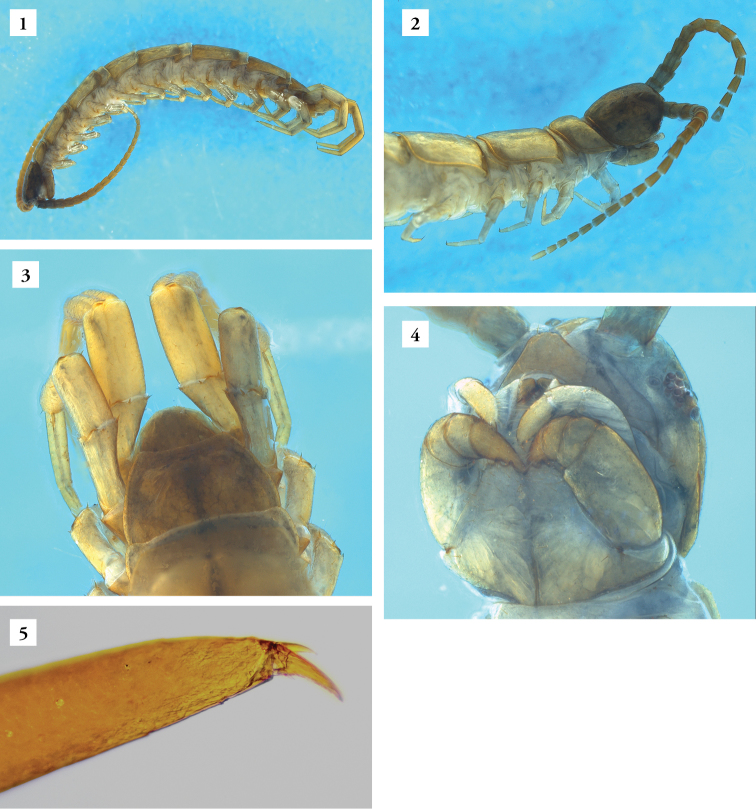
*Lithobiusproximus* Sseliwanoff, 1878, male (23 May–12 Sep 2017, Stand 4a): **1** total habitus, lateral view **2** anterior part of the body with a darker brownish to brown colour of the head and antennal articles, dorsolateral view **3** posterior part of the body, ultimate male legs with dorsal groove on femur and tibia (arrows), dorsal view **4** head in ventrolateral view **5** distal end of ultimate leg with apical claw and accessory apical claw. Photos by P. Ślipiński (**1–4**) and M. Romański (**5**).

**Figures 6–8.**
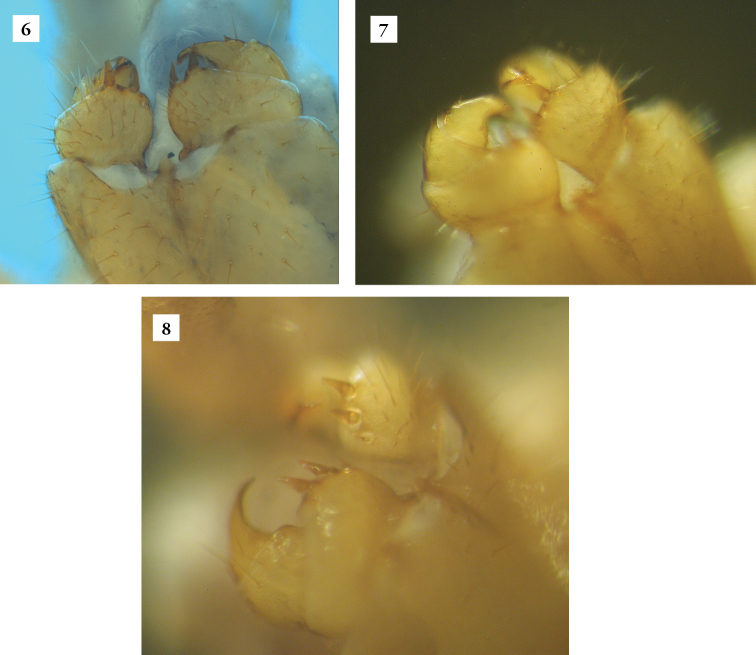
*Lithobiusproximus* Sseliwanoff, 1878: **6, 7** female gonopods with 2 + 2 spurs (23 May–12 Sep 2017, Stands 4b and 5) **8** female gonopods with 3 + 3 spurs marked by arrows (23 May–12 Sep 2017, Stand 5); Photos by P. Ślipiński (**6**) and J. Wytwer (**7, 8**).

*Lithobiusproximus* is formally treated as belonging to the subgenusEzembius Chamberlin, 1919 (Farzalieva and Esyunin 2008, Nefediev et al. 2017a, b, Nefediev et al. 2018). Species included in the subgenusEzembius are characterized by the number of their antennal articles being limited to about 20, similar to the members of the subgenusMonotarsobius Verhoeff, 1905, but they differ from them in that the tarsal articulation of the legs 1−13 is distinct, as in the subgenusLithobius Leach, 1814 (Eason 1974, 1976; Zapparoli and Edgecombe 2011). Therefore, it is easy to distinguish representatives of the genus *Ezembius* from the majority of centipedes inhabiting the litter of Central European forests, most often belonging to either subgenusLithobius or *Monotarsobius*. The morphology of specimens recorded in the Wigry National Park corresponds to the characteristics given by Zalesskaja (1978) as well as Farzalieva and Esyunin (2008). They are distinguished by having elongated antennal articles and a darker brownish to brown colour head compared to the rest of the body (Figures 1, 2). Marginal ridges of tergites 9, 11 and 13 are rather rounded, but tergites 13 and 15 occur with rounded gentle posterior projections. Males have a dorsal groove on the femur and tibia (Figure 3) and often also on the first tarsal segment of the ultimate legs. Females have gonopods with simple claws (Figures 6, 7), with 2 + 2 (most common) and sometimes with 2 + 3 or 3 + 3 spurs (Figure 8). However, in our examined material, the third spur (the most inner) was very small (< one-third of the first one), a feature that distinguishes it from Lithobius (Ezembius) sibiricus Gerstfeldt, 1858, whose males, moreover, have no sexual characters on the fifteenth pair of legs (Zalesskaja 1978, Farzalieva and Esyunin 2008). Other key features observed on the sampled specimens were also consistent with the redescription made by Farzalieva and Esyunin (2008), i.e. head with 9–10 ocelli in 3–4 rows, coxosternum with 2 + 2 sharp teeth (Figure 4), presence of the accessory apical claw on ultimate legs in both sexes (Figure 5) and the spinulation pattern on the legs.

Female specimens with 2 + 3 or 3 + 3 spurs on gonopods were treated as very rare (Farzalieva and Esyunin 2008) or aberrant (Nefediev et al. 2017a). Overall, in our material the ‘aberrant’ specimens accounted for 11% of all females.

#### Distribution.

**Figure 9.**
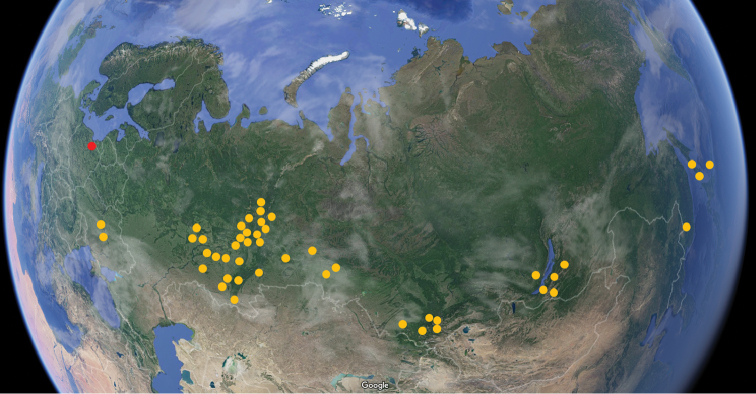
Distribution map of *Lithobiusproximus* Sseliwanoff, 1878 based on a summarisation of the published data (yellow dots; Zalesskaja 1978, Zalesskaja and Golovatch 1996, Chornyi and Kosyanenko 2003, Farzalieva and Esyunin 2008, Kosyanenko and Chornyi 2008, Kunakh 2013, Sergeeva 2013, Nefediev et al. 2017a, b, Nefediev et al. 2018) and our records (red dot; this paper).

*Lithobiusproximus* is the only representative of the subgenusEzembius in Poland. *Ezembius* was regarded as a subgenus of the genus *Lithobius* by Eason (1974) for the group that occurred in eastern and northern Asia. *Lithobiusproximus* was originally described from Irkutsk by Sseliwanoff (1878). Zalesskaja (1978) designated it as a Siberian species and later (Zalesskaja and Golovatch 1996) defined it as a centipede that inhabited the belt from the taiga to the steppe and suggested that the Volga River limited its spread to the west. This opinion was later repeated by Dyachkov (2017). Recently, this species was characterised as a widespread Siberian boreal species (Nefediev et al. 2017a), and later it was judged as a Eurasian species widely distributed in Russia, specifically in the Altai area ranging from the taiga on the lake shore up to the mountain tundra at approximately 2200 m a.s.l. (Nefediev et al. 2017b). Subsequently, Nefediev et al. (2018), referred to this species as an eastern European–Transsiberian temperate range species that occurred from the eastern Russian Plain (Republics of Mari El and Tatarstan, Kirov and Samara areas; i.e. respecting the Volga River line) in the west through Siberia to the Russian Far East (Maritime Province, Sakhalin and the Kuril Islands). However, *Lithobiusproximus* was also repeatedly recorded in Ukraine, from the Kanev Nature Reserve on the Dnieper Lowland (Chornyi and Kosyanenko 2003, Kosyanenko and Chornyi 2008) and from the ‘Chernyi Les’ forest near Kirovograd on the Dnieper Upland (Kunakh 2013). Both stands are in the forest-steppe belt, and the nearest stand in the Kanev Nature Reserve is over 750 km in a straight line from the Wigry National Park in Poland. Thus, our records represent the western most points of the entire *Lithobiusproximus* distribution area (Figure 9).

#### Ecology.

The present data indicate that *Lithobiusproximus* is neither an accessory nor an accidental species in the litter centipede community of the horn-beam forests in the Wigry National Park; rather, it is well anchored as a co-dominant species. Quantitative data on the epigeic fauna based on the extensive pitfall trapping proved that *Lithobiusproximus* co-dominates with two other lithobiomorph centipedes. The first is the common, eurytopic species Lithobius (Lithobius) forficatus Linnaeus, 1758, with a Holarctic range of distribution, and the second is the common forest species Lithobius (Monotarsobius) curtipes (C. Koch, 1847), with a Palearctic range. Our data appear to agree with phenological observations made by Farzalieva and Esyunin (2014) in the southern taiga of the Perm Cis-Ural region, where *Lithobiusproximus* was the most numerous species during summer. Similarly, Sergeeva (2013) recorded *Lithobiusproximus* as the second most frequent lithobiomorph species in the valley of Irtysh River, West Siberian region. In our observations, 20–30% of all centipedes caught by trapping during the summer, and a negligible amount during the “winter” (i.e. from September to May) sampling period, were found in both sampling seasons.

## Discussion

Geographically, the northeastern most edge of Poland is the most western part of the East European Plain (Russian Plain), the megaregion that is characterised by a classic latitudinal nature zonation combined with an increasing longitudinal continental character. Climate shifts result in the East European Plain to be covered by belts of biomes arranged from the tundra to the taiga, mixed coniferous-deciduous forests, broadleaved forests, steppe and semideserts to deserts in the south. Previous research has demonstrated that climate is the main factor that influences distribution of some soil fauna in the East European Plain (Chernov 1975, Penev 1992, Esjunin et al. 1995, Wytwer et al. 2009). On the other hand, some groups of soil animals seem to be subordinate to local conditions, as confirmed for earthworms (Penev et al. 1994a, b). Hence, there are many soil invertebrates where their actual distribution range would cover several geographical zones if only the local conditions allowed them to survive. *Lithobiusproximus* seems to be such an example. Zalesskaja and Golovatch (1996) reported that this centipede species occurs from the taiga to the steppe, although they supposed its spread to the west was limited by the Volga River. The Ukrainian data from the Dnieper Lowland (Chornyi and Kosyanenko 2003, Kosyanenko and Chornyi 2008) and Dnieper Upland (Kunakh 2013), and the present data from Poland, have changed our view about the actual range for this species.

Based on the known published data, *Lithobiusproximus* seems to be a species with high plasticity with regards to environmental requirements. Farzalieva and Esyunin (2008), in their review of centipedes of the Ural and Cis-Ural Area, noted a range of environments where it can survive, mainly different types of forests (spruce, pine, birch, oak and lime), and mostly wetlands, as well as other environments including gypsum quarry. The same authors (Farzalieva and Esyunin 2014) later examined the structure and seasonal dynamics of myriapods in the Perm Cis-Ural region, and classified this centipede as a forest species, i.e., a eurybiont and forest-preferring species that was most abundant in all forest and derivative habitats. Nefediev et al. (2017a) discussed data from Siberia and noted that this species tended to dwell in small-leaved forest stands. Our current research in the Wigry National Park suggests that *Lithobiusproximus* occurs almost exclusively in the oak-hornbeam forests, where in the summer it is one of the three dominant lithobiomorph centipedes inhabiting litter layers. Based on our observations, this species may be associated with deciduous forests much more than was previously thought.

## Supplementary Material

XML Treatment for Lithobius (Ezembius) proximus
